# Non-Surgical Causes of Death in the Emergency Department: A Five-Year Monocentric Clinicopathological Study

**DOI:** 10.3390/medicina62020293

**Published:** 2026-02-02

**Authors:** Adrian-Iosif Moldoveanu, Diana Maria Orzata, Gabriel Veniamin Cozma, Radu Gheorghe Dan, Ovidiu Alexandru Mederle, Flavia Zara

**Affiliations:** 1 Doctoral School, “Victor Babeș” University of Medicine and Pharmacy, 30041 Timisoara, Romania; adrian.moldoveanu@umft.ro (A.-I.M.); diana.orzata@umft.ro (D.M.O.); 2 Department of Surgery I, “Victor Babeș” University of Medicine and Pharmacy, 30041 Timisoara, Romania; gabriel.cozma@umft.ro; 3 Center for Hepato-Biliary-Pancreatic Surgery, “Victor Babeș” University of Medicine and Pharmacy, 30041 Timisoara, Romania; 4 Emergency Discipline, Department of Surgery, “Victor Babeș” University of Medicine and Pharmacy, 30041 Timisoara, Romania; mederle.ovidiu@umft.ro; 5 Department of Surgery, Multidisciplinary Centre for Research, Evaluation, Diagnosis and Therapies in Oral Medicine, “Victor Babeș” University of Medicine and Pharmacy, 30041 Timisoara, Romania; 6 Department of Microscopic Morphology, “Victor Babeș” University of Medicine and Pharmacy, 30041 Timisoara, Romania; flavia.zara@umft.ro; 7 Department of Pathology, Emergency City Hospital, 300254 Timisoara, Romania

**Keywords:** non-surgical mortality, emergency department, autopsy, clinicopathological concordance, sepsis, cardiovascular pathology, COVID-19

## Abstract

*Background and objectives*: Non-surgical deaths in the Emergency Department (ED) occur in the context of severe acute pathology and frequently under conditions of limited diagnostic time and incomplete clinical information. Data integrating ante-mortem clinical assessment with medico-legal autopsy results remain scarce, particularly in Central and Eastern Europe. *Materials and Methods*: We conducted a retrospective, monocentric descriptive clinicopathological study including 45 consecutive non-surgical deaths occurring in the Emergency Department of a tertiary care hospital between January 2019 and December 2023. Clinical, biological, and temporal data were retrospectively analyzed and correlated with complete medico-legal autopsy findings in order to establish the cause of death and to assess clinicopathological concordance. *Results:* The mean patient age was 74.3 years, and the median time from ED admission to death was 142 min. Cardiovascular disease was the most frequent cause of death in this cohort (35.6%), followed by sepsis (22.2%), non-COVID respiratory causes (15.6%), and SARS-CoV-2 infection (17.8%). Complete clinicopathological concordance was observed in 37.8% of cases, while partial concordance predominated (57.8%). Total discordance was rare (4.4%). Autopsy findings frequently demonstrated multisystem involvement, particularly in deaths attributed to sepsis and COVID-19. *Conclusions*: In this descriptive, autopsy-based cohort, non-surgical deaths in the Emergency Department were associated with advanced disease severity and rapid clinical deterioration, limiting complete etiological clarification prior to death. The high rate of partial clinicopathological concordance may reflect the complexity of terminal pathophysiological mechanisms encountered in emergency settings. Systematic clinicopathological correlation through autopsy remains essential for understanding selected cases of acute non-surgical mortality in selected, rapidly fatal ED cases.

## 1. Introduction

Non-surgical deaths occurring in the Emergency Department (ED) represent a major challenge for emergency medicine and have been described as a marker of extreme disease severity rather than a distinct clinical entity. Patients presenting with acute medical conditions often have non-specific symptoms, rapidly evolving clinical instability, and frequent multisystem involvement. Under these circumstances, diagnostic evaluation is constrained by time pressure and limited clinical information, which may restrict the possibility of establishing a complete etiological diagnosis before death.

Cardiovascular diseases remain the leading cause of acute mortality worldwide and account for a substantial proportion of deaths occurring in the ED. Acute myocardial infarction, cardiogenic shock, and malignant arrhythmias are associated with high early mortality, particularly in elderly patients and in those with advanced comorbidities. Several studies have shown that a significant number of cardiovascular deaths occur before disease-specific therapies can be fully implemented, reflecting delayed presentation and advanced biological severity at the time of ED admission [1,2,3]. In this context, several studies have suggested that Emergency Department mortality is often associated with late-stage disease trajectories rather than isolated, unpredictable acute events.

Sepsis and septic shock constitute another major cause of non-surgical mortality in emergency settings. Despite the adoption of standardized diagnostic criteria through the Sepsis-3 definitions, sepsis-related mortality remains high, especially during the early phase of presentation. This period is characterized by diagnostic uncertainty and rapid progression toward multiple organ dysfunction. Previous studies have demonstrated a strong association between early mortality and the severity of organ dysfunction at presentation, as reflected by SOFA scores and biological markers such as lactate levels [4,5,6]. These observations underline the dynamic and heterogeneous nature of sepsis, a characteristic that has been associated with limitations in early etiological clarification in emergency settings.

Acute respiratory failure is also a frequent cause of death in the ED and is commonly associated with severe pneumonia, acute respiratory distress syndrome (ARDS), or acute exacerbations of chronic pulmonary diseases. Severe hypoxemia, acidosis, and hypercapnia at presentation have been identified as independent predictors of mortality, particularly when respiratory failure is accompanied by sepsis or hemodynamic instability [7,8,9]. In such rapidly evolving clinical scenarios, Emergency Department assessment is frequently syndromic by necessity.

The COVID-19 pandemic has further modified the etiological spectrum and pathological characteristics of non-surgical deaths in the ED. Autopsy studies have demonstrated that SARS-CoV-2 infection is associated not only with diffuse alveolar damage but also with widespread endothelial injury, pulmonary microthrombosis, and systemic vascular involvement. These findings highlight the complex pathological substrate underlying early mortality in severe COVID-19 [10,11,12]. However, most available data originate from large autopsy series or intensive care cohorts, with limited focus on early ED deaths.

In this context, autopsy remains the reference standard for determining the cause of death, particularly in emergency settings where ante-mortem diagnostic assessment is often incomplete. Numerous studies continue to report discrepancies between clinical diagnoses and post-mortem findings, even in modern medical practice. These discrepancies are especially frequent in cases of sepsis, pulmonary embolism, atypical myocardial infarction, and previously undiagnosed malignant disease [13,14,15]. Integrating clinical, biological, and anatomopathological data has been shown to provide valuable insight into the mechanisms of death, particularly in settings characterized by limited ante-mortem diagnostic information.

Most available data on Emergency Department mortality originate from North America and Western Europe. In contrast, studies from Central and Eastern Europe remain scarce and are predominantly monocentric. This is particularly relevant given the higher burden of cardiovascular disease and increased rates of premature mortality reported in Eastern European populations [16,17]. Consequently, descriptive clinicopathological studies from this region may contribute complementary insights, while acknowledging inherent limitations related to sample size and study design.

The present study was designed as a retrospective, monocentric, descriptive and exploratory clinicopathological analysis of non-surgical deaths occurring in the Emergency Department, integrating ante-mortem clinical data with complete medico-legal autopsy findings. The aim was not to evaluate diagnostic performance or system efficiency, but to describe causes of death and patterns of clinicopathological concordance in a selected cohort of rapidly fatal cases.

## 2. Materials and Methods

A retrospective, descriptive, exploratory, monocentric study was performed, including all non-surgical deaths that occurred in the Emergency Department of Emergency City Hospital, a tertiary care university-affiliated hospital in Timișoara, Romania, over a five-year period, between January 2019 and December 2023. All cases were included consecutively, within the limits of availability of complete clinical and medico-legal documentation, without additional selection criteria. Because inclusion required the availability of complete medico-legal autopsy reports, the study cohort represents a selected subset of Emergency Department deaths and may not be representative of overall ED mortality.

Adult patients who died in the Emergency Department as a result of an acute non-surgical medical condition were eligible for inclusion. Non-surgical death was defined as death occurring in the absence of a primary indication for emergency surgical intervention at presentation. Patients with conditions requiring immediate surgical management, such as acute surgical abdomen, acute mesenteric ischemia, gastrointestinal perforation, or other surgical emergencies, were excluded. Deaths related to trauma were also excluded. In addition, cases of acute intoxication without an identifiable underlying medical pathology and cases with incomplete clinical or anatomopathological documentation were not included in the analysis.

Clinical data were collected retrospectively from Emergency Department medical records. These included demographic characteristics, mode of presentation, vital signs at admission, and the clinical course during the Emergency Department stay. Available paraclinical investigations performed at presentation were reviewed, including routine laboratory tests, inflammatory and cardiac biomarkers, and arterial blood gas analysis, when available. Imaging studies obtained in the Emergency Department were also reviewed when they contributed to the initial clinical assessment.

Complete medico-legal autopsy reports were available for all included cases. Macroscopic and microscopic findings were reviewed and correlated with clinical and biological data in order to establish the cause of death. Clinicopathological concordance was assessed using a pragmatic, descriptive classification, categorizing cases as complete concordance, partial concordance, or discordance, based on the level of agreement between the dominant ante-mortem clinical diagnosis and post-mortem findings. Complete concordance was defined as full agreement between the dominant clinical diagnosis established in the Emergency Department and the autopsy-determined cause of death. Partial concordance referred to cases in which the major clinical syndrome was correctly identified ante mortem, but additional or contributory pathological mechanisms were established only at autopsy. Discordance was defined as a lack of agreement between the primary clinical diagnosis and the autopsy-determined cause of death. This classification was applied for descriptive purposes and was not intended as a validated measure of diagnostic performance.

Based on clinicopathological correlation, cases were classified into the main categories of non-surgical causes of death, including cardiovascular pathology, sepsis with or without multiple organ dysfunction, respiratory causes, and advanced oncological disease. Sepsis was defined according to Sepsis-3 criteria, based on the presence of suspected or confirmed infection associated with acute organ dysfunction. For descriptive purposes, cases presenting with single-organ dysfunction at Emergency Department admission were classified as sepsis without established multiple organ dysfunction, whereas cases with documented involvement of multiple organ systems were classified as sepsis with multiple organ dysfunction. During the COVID-19 pandemic period, cases associated with SARS-CoV-2 infection were identified based on RT-PCR confirmation and/or a characteristic clinical and anatomopathological presentation consistent with COVID-19.

Data analysis was descriptive. Continuous variables were expressed as means and ranges, while categorical variables were presented as absolute numbers and percentages. Given the sample size and the descriptive aim of the study, no inferential statistical analyses were performed, and quantitative findings were interpreted strictly as observational descriptions without comparative intent.

Patient admission and medical management in the Emergency Department were carried out under the principle of implied consent, applicable in life-threatening situations. The study was retrospective and based on anonymized data derived exclusively from deceased patients; therefore, informed consent was not required. The study protocol was approved by the institutional ethics committee and was conducted in accordance with the principles of the Declaration of Helsinki.

## 3. Results

### 3.1. Study Cohort Characteristics

Between January 2019 and December 2023, 45 consecutive cases of non-surgical deaths occurring in the Emergency Department and meeting the predefined inclusion criteria were included. The mean age was 74.3 years (range 49–102 years). There were 27 male patients (60%) and 18 female patients (40%). Regarding residence, 24 patients (53.3%) were from urban areas and 21 (46.7%) from rural areas.

### 3.2. Mode of Presentation and Time to Death in the Emergency Department

Most patients were brought to the Emergency Department by emergency medical services (41 cases, 91.1%). Four patients (8.9%) presented by their own means. Time from admission to death ranged from 22 to 478 min. The median time to death was 142 min, with an interquartile range of 93–234 min. The demographic data and mode of presentation are summarized in Table 1.

### 3.3. Etiological Distribution of Non-Surgical Deaths

Based on clinicopathological correlation between the dominant Emergency Department diagnosis and complete medico-legal autopsy findings, non-surgical causes of death were classified into five main etiological categories.

Cardiovascular pathology was identified in 16 cases (35.6%). Sepsis, with or without established multiple organ dysfunction at the time of Emergency Department presentation, accounted for 10 cases (22.2%). Respiratory causes unrelated to COVID-19 were identified in 7 cases (15.6%). Eight deaths (17.8%) were associated with SARS-CoV-2 infection and were analyzed as a distinct subgroup. Advanced oncological disease was identified as the cause of death in 4 cases (8.8%).

For each etiological group, the dominant clinical diagnosis formulated in the Emergency Department was correlated with the main autopsy findings. A synthetic overview of this correlation is provided in Table 2.

### 3.4. Clinical and Laboratory Results

Clinical and laboratory findings were analyzed descriptively and are presented in relation to the main etiological categories established through clinicopathological correlation.

In cases attributed to cardiovascular pathology, cardiac biomarkers showed elevated values at presentation. Troponin I values ranged from 12 to 45 ng/mL, while NT-proBNP levels ranged from 10,000 to 30,000 pg/mL. Electrolyte disturbances were frequently documented, particularly involving potassium, with values between 2.8 and 6.4 mmol/L.

In cases classified as sepsis biological abnormalities consistent with systemic infection and organ dysfunction were observed at Emergency Department presentation. Lactate levels ranged from 6.8 to 12 mmol/L. C-reactive protein values exceeded 240 mg/L, and procalcitonin levels ranged from 25 to 63 ng/mL. Leukocytosis was commonly present, with white blood cell counts between 24 and 38 ×10^9^/L.

In cases associated with SARS-CoV-2 infection, laboratory findings reflected a hyperinflammatory and procoagulant profile at presentation. Interleukin-6 levels ranged from 70 to 310 pg/mL. D-dimer values ranged from 3.2 to 12 µg/mL, and ferritin levels ranged from 1500 to 4200 ng/mL. Lymphopenia was consistently documented, with lymphocyte counts below 700/µL in all cases.

These clinical and laboratory findings represent values documented at the time of Emergency Department assessment and are reported descriptively, without comparative or predictive intent.

### 3.5. Cases Associated with SARS-CoV-2 Infection

Eight cases associated with SARS-CoV-2 infection were identified during the pandemic period (March 2020 to February 2021). These cases are presented as a distinct subgroup due to their specific clinicopathological characteristics, rather than for comparative analysis with other etiological categories. At Emergency Department presentation, patients predominantly exhibited acute respiratory failure, frequently accompanied by systemic manifestations of critical illness. Laboratory findings documented at presentation, detailed in Section 3.4, reflected a systemic inflammatory and procoagulant profile, consistent with severe SARS-CoV-2 infection.

Histopathological examination revealed diffuse alveolar damage (DAD), pulmonary microthrombosis, capillary endothelitis, fibrinous microthrombi, and hemorrhagic alveolitis. These findings indicate extensive pulmonary and vascular involvement and were not observed in non-COVID respiratory deaths included in this cohort.

Clinicopathological correlation indicates that, although Emergency Department assessment was primarily focused on respiratory manifestations, autopsy findings demonstrated multisystem involvement in these cases. These findings contributed to the etiological classification of SARS-CoV-2–associated deaths and to the descriptive characterization of their pathological substrate.

### 3.6. Clinicopathological Concordance

Clinicopathological concordance between the dominant Emergency Department diagnosis and the autopsy-established cause of death was complete in 17 cases (37.8%), partial in 26 cases (57.8%), and discordant in 2 cases (4.4%) (Table 3).

Complete concordance was most frequently observed in cases of advanced oncological disease and in a subset of cardiovascular deaths, where the primary clinical diagnosis formulated in the Emergency Department fully corresponded to the autopsy findings. Partial concordance predominated in cases of sepsis, respiratory causes, and COVID-19–related deaths, in which the major clinical syndrome was recognized ante mortem, while the full extent of pathological mechanisms was established only at autopsy.

Clinicopathological discordance was identified in two cardiovascular cases. In one case, the ante-mortem clinical diagnosis suggested sepsis, whereas autopsy established acute pulmonary thromboembolism as the primary cause of death. In the second case, no acute ischemic cardiac event was suspected clinically, while autopsy revealed a recent myocardial infarction (24–48 h) associated with acute cardiac failure; an undiagnosed renal malignancy was identified as a contributory condition.

### 3.7. Autopsy Findings

Complete medico-legal autopsy was performed in all included cases. Autopsy findings were analyzed descriptively and are presented according to the main etiological categories established for the study cohort, with representative cases illustrating different patterns of clinicopathological concordance.

**Figure 1:**
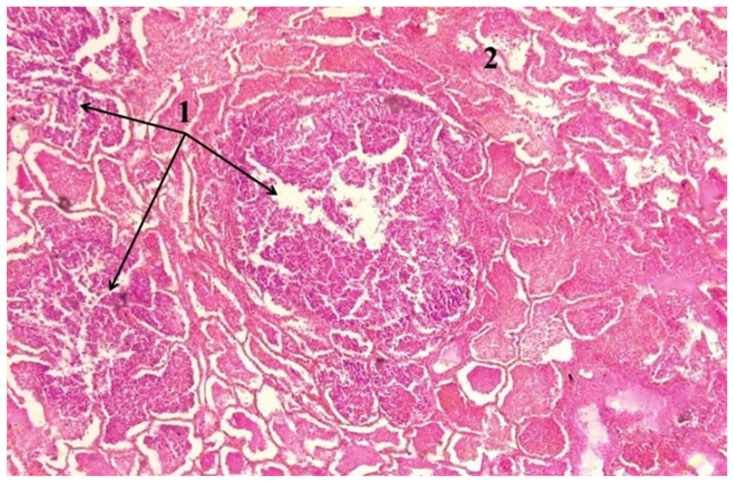
Histopathological features of acute myocardial infarction. Hematoxylin and eosin–stained myocardial section showing (1) an area of acute myocardial infarction characterized by coagulative necrosis of cardiomyocytes, marked hypereosinophilia, loss or pyknosis of nuclei, interstitial edema, and early acute inflammatory infiltrate, consistent with recent ischemic injury, and (2) adjacent myocardial tissue with relatively preserved architecture and viable cardiomyocytes.

In cases attributed to cardiovascular pathology, autopsy most frequently identified extensive acute or subacute myocardial infarction. Additional findings included myocardial rupture, acute fibrinous pericarditis, papillary muscle involvement, and features of chronic ischemic heart disease. In several cases, the extent and localization of myocardial injury documented at autopsy exceeded the abnormalities identified during Emergency Department evaluation. Figure 1 illustrates a cardiovascular death with partial clinicopathological concordance, in which Emergency Department assessment identified a clinical presentation consistent with acute coronary syndrome, while autopsy documented the extent and localization of an acute myocardial infarction involving the left ventricular wall.

**Figure 2:**
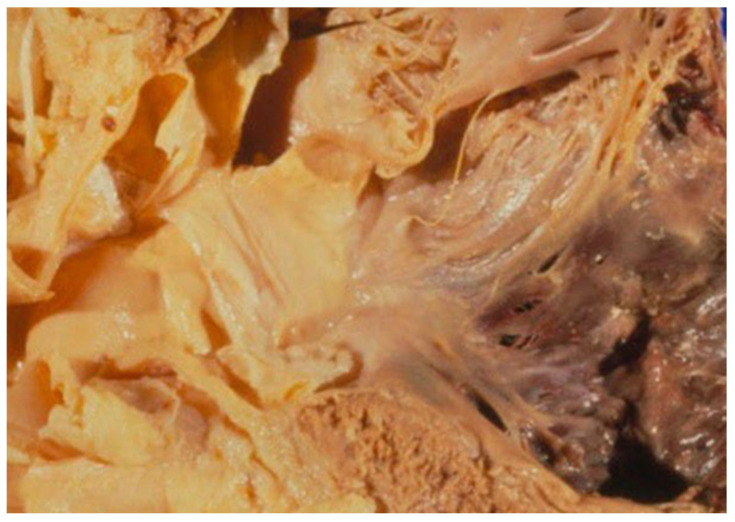
Acute fibrinous pericarditis complicating acute myocardial infarction. Gross pathological appearance of the pericardial surface showing dull, irregular serosal surfaces covered by fibrinous, filamentous exudate, consistent with acute fibrinous pericarditis. This finding represents an early inflammatory reaction following acute myocardial infarction.

Figure 2 presents another cardiovascular case with partial clinicopathological concordance, in which Emergency Department evaluation suggested myocardial ischemia, whereas autopsy revealed acute ischemic myocardial injury associated with fibrinous pericarditis, representing additional pathological findings not identified ante mortem.

**Figure 3:**
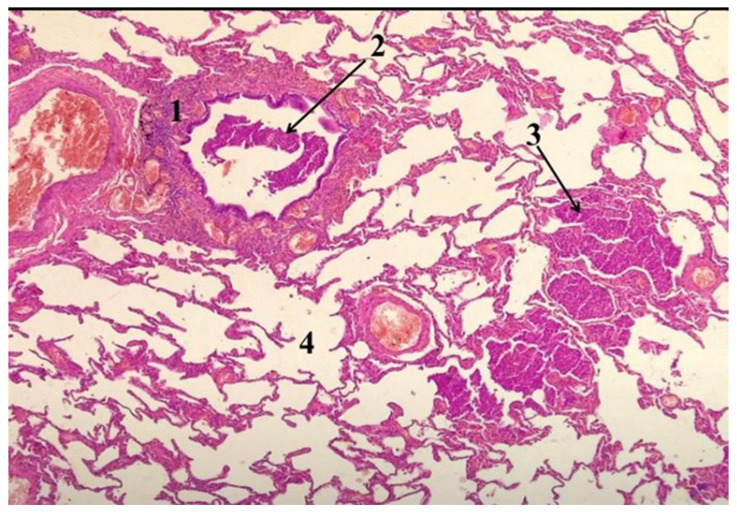
Histopathological pulmonary findings in non-surgical death. Hematoxylin and eosin–stained lung section demonstrating (1) a pulmonary vessel with intraluminal thrombus consistent with recent or partially organized pulmonary thrombosis, (2) a bronchiole with relatively preserved wall structure, (3) an area of alveolo-interstitial inflammatory consolidation suggestive of acute pneumonia or early acute respiratory distress syndrome, and (4) dilated alveolar spaces with septal thinning consistent with pulmonary emphysema.

In non-COVID respiratory deaths, autopsy findings included bronchopneumonia, diffuse alveolar damage, pulmonary edema, and pulmonary embolism. The distribution and severity of pulmonary pathology varied among cases. Figure 3 illustrates a respiratory death with partial clinicopathological concordance, in which Emergency Department presentation was dominated by acute respiratory failure, while autopsy identified pulmonary thrombotic and alveolo-interstitial inflammatory pathology contributing to the cause of death.

In cases associated with SARS-CoV-2 infection, autopsy demonstrated diffuse alveolar damage, pulmonary microthrombosis, endothelial injury, and alveolar hemorrhage. In several cases, extrapulmonary pathological findings were also documented, including cardiac and renal involvement.

In cases attributed to advanced oncological disease, autopsy confirmed disseminated malignant disease, with widespread metastatic involvement and organ failure consistent with advanced-stage cancer. In these cases, clinicopathological concordance was complete.

## 4. Discussion

Non-surgical mortality in the Emergency Department (ED) may reflect the interaction between the biological severity of acute disease and the intrinsic limitations of emergency evaluation, particularly in rapidly fatal cases. Patients who die shortly after presentation often exhibit extreme physiological instability, incomplete clinical information, and a very limited time window for diagnostic clarification. Within this descriptive and autopsy-based context, ED mortality should be interpreted cautiously and may reflect advanced disease trajectories rather than as an indicator of diagnostic or therapeutic failure.

Recent epidemiological data confirm the central role of cardiovascular disease, sepsis, and respiratory failure in Emergency Department mortality and indicate that these syndromes frequently coexist and overlap, complicating early clinical assessment and precise etiological differentiation [18,19]. In this context, early risk stratification in the Emergency Department relies on clinical triage tools such as qSOFA or NEWS; however, their prognostic accuracy remains limited in early presentations and does not fully capture biological heterogeneity, particularly in sepsis [20]. In the present cohort, qSOFA components were frequently documented as part of routine clinical assessment; however, qSOFA scores were not systematically recorded in a standardized manner and were therefore not analyzed as a separate variable. Moreover, emerging data suggest that distinct biological phenotypes are associated with markedly different early mortality trajectories, which may further limit the predictive value of initial clinical assessment in the Emergency Department [6].

### 4.1. Cardiovascular Mortality: Acute Event Versus Terminal Decompensation

In the present cohort, cardiovascular pathology represented the most frequent cause of non-surgical death in the ED. Clinicopathological correlation suggests that, in many cases, death occurred on the background of advanced chronic cardiovascular disease with acute decompensation rather than as the result of an isolated, unpredictable acute event.

This observation is consistent with published data indicating that patients with cardiogenic shock or acute coronary syndromes often present late, at a stage of severe hemodynamic compromise, frequently associated with established multiorgan dysfunction [3,21]. In such situations, the potential impact of therapeutic interventions initiated in the ED may be limited by the advanced biological severity already present at admission.

The markedly elevated myocardial necrosis and ventricular stress markers observed in this study are compatible with the presence of extensive cardiac injury at presentation. Elevated troponin and NT-proBNP levels in acute settings do not necessarily indicate a classical type 1 myocardial infarction but may reflect global myocardial injury secondary to hypoxia, sepsis, respiratory failure, or profound metabolic disturbances [22]. This pathophysiological overlap contributes to the difficulty of precise etiological classification in the ED and may explain the high proportion of partial clinicopathological concordance.

Autopsy findings indicate that, while the dominant cardiovascular syndrome is usually recognized clinically, the terminal mechanism of death—such as malignant arrhythmia, extensive ischemic injury, or global myocardial dysfunction—is often clarified only post mortem. Previous studies have emphasized that cardiogenic shock frequently represents the final stage of a complex cascade in which acute ischemia overlaps with a chronic structural substrate, making ante-mortem differentiation difficult, particularly in terminal phases [3,23].

Taken together, these findings support the interpretation that cardiovascular mortality in the ED frequently reflects terminal decompensation of pre-existing disease rather than sudden, isolated acute events. This interpretation aligns with data showing that reductions in acute cardiovascular mortality depend largely on early recognition and intervention before the terminal phase, often outside the hospital setting [24].

### 4.2. Sepsis: Limitations of Initial Assessment in a Dynamic Syndrome

Sepsis represented a major cause of non-surgical mortality in the present cohort. Although the septic syndrome was clinically suspected in most cases, clinicopathological analysis suggests that the full extent of organ involvement and the biological trajectory of the disease were often difficult to fully appreciate at presentation.

Sepsis is increasingly recognized as a dynamic and heterogeneous syndrome rather than a single acute event. Its evolution involves rapidly changing inflammatory, immunological, and metabolic processes [25]. In this context, initial Emergency Department evaluation often captures only a snapshot of this evolving process, which may limit the ability to predict progression toward irreversible multiorgan failure and may help explain the predominance of partial clinicopathological concordance observed in septic deaths.

The severe hyperlactatemia and markedly elevated inflammatory markers observed at presentation are consistent with advanced systemic involvement. Hyperlactatemia reflects not only tissue hypoperfusion but also profound metabolic dysregulation and has been shown to be strongly associated with early mortality, even when appropriate treatment is initiated promptly [26]. These findings support the interpretation that many sepsis-related deaths in the Emergency Department occur after a critical biological threshold may have already been crossed prior to hospital arrival.

Clinical scoring systems used in Emergency Department triage are useful for identifying high-risk patients but have limited sensitivity in early sepsis and do not fully capture the biological heterogeneity of the syndrome [20,27]. Moreover, recent evidence supports the existence of distinct sepsis phenotypes associated with markedly different early mortality trajectories, independent of the timeliness of standard treatment, further limiting the prognostic value of initial clinical assessment [6].

### 4.3. Time to Death: An Objective Diagnostic Constraint in the ED

A central finding of this study is the short interval between ED admission and death, with a median time of 142 min. Previous studies have reported that patients who die early in the ED constitute a distinct subgroup characterized by extreme disease severity and limited opportunity for diagnostic refinement [18].

Literature on diagnostic discrepancies consistently indicates that extreme time pressure, severe instability, and incomplete data are associated with an increased likelihood of differences between ante-mortem and post-mortem diagnoses, particularly in sepsis, cardiogenic shock, and severe respiratory failure [28,29]. In this context, many discrepancies identified at autopsy would be unlikely to influence immediate management in the ED.

### 4.4. COVID-19: Autopsy Confirmation of a Systemic Disease

In the analyzed cohort, deaths associated with SARS-CoV-2 infection constituted a distinct clinical and clinicopathological subgroup. Although ED presentation was dominated by acute respiratory failure, autopsy data indicate that the mechanisms of death in severe COVID-19 often extend beyond the paradigm of viral pneumonia or acute respiratory distress syndrome, supporting the interpretation of COVID-19 as a systemic disease process [10,11,30,31,32].

Published autopsy studies have consistently reported diffuse vascular involvement characterized by endothelitis, microthrombosis, and hemostatic dysregulation at both pulmonary and extrapulmonary levels [10,12,30]. These pathological features have been associated with the rapidly progressive and often disproportionate clinical deterioration relative to initial radiological findings, which may limit the ability of ED assessment to anticipate the terminal mechanism of death [11,33].

Clinicopathological analysis suggests that initial ED evaluation frequently captures only the dominant clinical expression of the disease, while the true extent of systemic involvement may remain difficult to assess at presentation. Literature identifies SARS-CoV-2–induced endothelial dysfunction as a central mechanism of severe disease, leading to microcirculatory disturbances and tissue ischemia with rapid progression to multiorgan failure, independent of the severity of respiratory failure [30,31,34].

A relevant aspect identified in this cohort is the overlap of COVID-19 with other critical syndromes frequently encountered in the ED, such as sepsis or cardiovascular collapse. Although these cases may be clinically classified syndromically, autopsy findings often demonstrate a multifactorial mechanism of death involving severe coagulopathy, endothelial injury, and concurrent multiorgan dysfunction [12,32,35]. This overlap may explain the difficulty of precise ante-mortem etiological classification and supports the analytical consideration of COVID-19 deaths as a distinct entity in ED mortality studies.

Overall, clinicopathological data from the present analysis support the interpretation that COVID-19–associated deaths in the ED represent the final expression of a complex systemic disease, and that autopsy continues to play an essential role in understanding the true mechanisms of death, particularly in the context of early mortality and the temporal constraints inherent to emergency medicine [11,33,36].

### 4.5. Clinicopathological Concordance: An Expression of Clinical Complexity

Analysis of clinicopathological concordance is a classical tool for evaluating diagnostic accuracy; however, its interpretation requires a nuanced approach in the context of emergency medicine. In the present study, complete concordance was observed in a limited number of cases, while partial concordance predominated and total discordance was rare, which may reflect the biological complexity of critically ill patients and the temporal constraints of ED evaluation.

Contemporary literature shows that discrepancies between ante-mortem diagnoses and causes of death persist even under modern care conditions, with most discrepancies relating to mechanisms rather than diagnostic direction [37,38]. In this context, partial concordance—correct identification of the dominant syndrome without full characterization of the terminal mechanism—represents a frequent and clinically explainable outcome in acute settings.

In emergency medicine, diagnosis is predominantly syndromic and prioritizes patient stabilization. In rapidly fatal cases, identification of cardiovascular collapse, sepsis, or respiratory failure is clinically feasible, whereas fine etiological delineation of terminal mechanisms is often achievable only post mortem through anatomopathological examination [29].

The results of this study are consistent with published data indicating a higher frequency of clinicopathological discrepancies in early deaths, characterized by limited time for investigations and diagnostic refinement, particularly in sepsis, cardiogenic shock, and severe respiratory failure [39]. In this framework, partial concordance may reflect the limits of initial assessment rather than true diagnostic error.

Literature on patient safety emphasizes that a significant proportion of discrepancies identified post mortem would not have altered immediate therapeutic management [28]. Therefore, autopsy remains a primary instrument for clinical integration and in-depth understanding of death mechanisms, contributing to a clearer comprehension of acute mortality complexity in the ED.

### 4.6. Conceptual Implications and Perspectives

The results of the present study suggest that non-surgical mortality in the Emergency Department (ED) should be interpreted as the expression of the interaction between biological disease severity, delayed presentation, and the constraints of acute clinical assessment, rather than as a direct indicator of diagnostic or therapeutic performance. Integrated analysis of causes of death, time to death, and clinicopathological concordance highlights the inherent limitations of emergency medicine when faced with biological trajectories that are already advanced at the time of presentation.

The data from this study support the need for a nuanced interpretation of ED mortality, particularly in rapidly fatal cases, where limited temporal intervals may restrict the possibility of complete etiological clarification or meaningful modification of clinical evolution. Contemporary literature indicates that, in such contexts, mortality predominantly reflects initial disease severity and underlying biology rather than deficiencies in medical care [18].

The findings also support the role of autopsy as an essential tool for clinical integration and understanding of terminal mechanisms of death. In accordance with recent literature, autopsy contributes primarily to diagnostic completion and clinical audit, without constituting a rigid standard for evaluating individual performance, especially in situations characterized by high biological complexity and rapid clinical evolution [29,37].

From a practical perspective, these observations suggest that reducing early mortality in the ED cannot be achieved solely by optimizing interventions during terminal phases. Literature demonstrates that early interventions along the continuum of care—early recognition of clinical deterioration, rapid access to medical services, and efficient referral to specialized care—have a more relevant impact on prognosis [40]. In this sense, ED mortality frequently reflects the final stage of a pathological process initiated prior to hospital presentation.

At the same time, the results underscore the need for a realistic approach to the concept of diagnosis in emergency medicine. Under conditions of extreme instability and temporal constraints, correct syndromic diagnosis and prompt initiation of appropriate treatment represent relevant clinical objectives, even if the final etiological mechanism is established subsequently. This approach is supported by literature on clinical uncertainty and decision-making in acute settings, which describes emergency medicine as a framework of probabilistic reasoning [41].

In conclusion, the implications of this study extend beyond the description of causes of death and propose an integrated perspective on non-surgical mortality in the ED, in which biological severity, time, and contextual limitations of acute assessment are central elements for realistic interpretation of mortality and for understanding the role of contemporary emergency medicine.

### 4.7. Study Limitations

This study has several limitations that should be considered when interpreting the results. The retrospective design and monocentric nature may limit the generalizability of the findings to other emergency departments. The relatively small sample size reflects the consecutive inclusion of non-surgical deaths with complete medico-legal autopsy and does not allow for inferential statistical analyses or broad generalization of the results. The analysis is limited to patients who died in the Emergency Department and does not include cases transferred to specialized wards, which may introduce selection bias. Additionally, the short interval between presentation and death in many cases restricted the availability of complete clinical data and may have influenced etiological classification. Therefore, the findings should be interpreted as descriptive and hypothesis-generating rather than directly generalizable to other Emergency Department populations. Despite these limitations, the integrated clinicopathological approach provides complementary insights into the mechanisms of non-surgical mortality in the Emergency Department.

## 5. Conclusions

Non-surgical mortality in the Emergency Department is frequently associated with extremely severe acute pathology, characterized by delayed presentation and rapid clinical deterioration. Under these conditions, complete etiological evaluation may not be achievable before death, and clinical management is necessarily based on a syndromic approach.

Cardiovascular disease and sepsis were the most frequent causes of death in this cohort. In many cases, death occurred in the background of advanced chronic disease or an already established systemic syndrome rather than as the result of an isolated acute event. Deaths associated with COVID-19 further illustrate the systemic nature of the disease and underscore the value of autopsy in clarifying complex mechanisms of death.

The predominance of partial clinicopathological concordance may reflect the biological complexity of critically ill patients and the temporal constraints inherent to emergency medicine. In this context, correct syndromic recognition and timely initiation of appropriate treatment represent realistic and clinically relevant objectives.

Overall, these findings support a nuanced interpretation of Emergency Department mortality that integrates disease severity, time to death, and the limitations of acute diagnostic assessment, while emphasizing the essential role of clinicopathological correlation in understanding non-surgical causes of death.

## Figures and Tables

**Table 1:** Demographic and clinical characteristics of the study population.

Characteristic	Value
Total number of cases, *n*	45
Age, mean (range), years	74.3 (49–102)
Sex—Male, *n* (%)	27 (60.0)
Sex—Female, *n* (%)	18 (40.0)
Area of residence—Urban, *n* (%)	24 (53.3)
Area of residence—Rural, *n* (%)	21 (46.7)
Mode of presentation—Emergency medical services, *n* (%)	41 (91.1)
Mode of presentation—Self-presentation, *n* (%)	4 (8.9)
Time to death in ED—Median (IQR), minutes	142 (93–234)
Time to death in ED—Range, minutes	22–478

**Table 2:** Etiological distribution of non-surgical deaths with clinicopathological correlation.

Etiology	Dominant ED Diagnosis	Main Autopsy Findings	Clinicopathological Concordance Pattern	No. of Cases (%)
Cardiovascular pathology	Acute coronary syndrome/cardiogenic shock	Extensive myocardial infarction, cardiomyopathy, malignant arrhythmias, pulmonary thromboembolism (PE)	9 Complete 5 Partial 2 Discordant	16 (35.6)
Sepsis ± multiple organ dysfunction	Suspected sepsis/septic shock	Septic focus with acute organ dysfunction	2 Complete 8 Partial	10 (22.2)
Respiratory causes (non-COVID)	Acute respiratory failure	Pneumonia, acute respiratory distress syndrome (ARDS), acute exacerbation of chronic pulmonary disease	2 Complete 5 Partial	7 (15.6)
COVID-19 (SARS-CoV-2 infection)	COVID-19 pneumonia	Diffuse alveolar damage (DAD) with pulmonary microthrombosis	8 Partial	8 (17.8)
Advanced oncological disease	Clinical deterioration in known or occult malignancy	Disseminated advanced malignancy	4 Complete	4 (8.8)

Cases with clinicopathological discordance are included within the etiological categories according to autopsy-established cause of death and are described separately in Section 3.6.

**Table 3:** Clinicopathological concordance between ante-mortem diagnosis and autopsy findings.

Type of Concordance	Number of Cases (*n*)	Percentage (%)
Complete concordance	17	37.8
Partial concordance	26	57.8
Discordant	2	4.4
Total	45	100

## Data Availability

The datasets used and analyzed during the current study are available from the corresponding author.
